# Production and Characterization of Antioxidant Properties of Exopolysaccharide(s) from *Peanibacillus mucilaginosus* TKU032

**DOI:** 10.3390/md14020040

**Published:** 2016-02-19

**Authors:** Tzu-Wen Liang, Shih-Chun Tseng, San-Lang Wang

**Affiliations:** 1 Life Science Development Center, Tamkang University, No. 151, Yingchuan Rd., Tamsui, New Taipei City 25137, Taiwan; ltw27@ms55.hinet.net; 2 Department of Chemistry, Tamkang University, New Taipei City 25137, Taiwan; 130805@mail.tku.edu.tw

**Keywords:** *Paenibacillus mucilaginosus*, exopolysaccharides, squid pen, culture conditions, antioxidant activity

## Abstract

Natural polysaccharides have received much attention due to their wide range of applications. Although most microbial exopolysaccharides (EPSs) use sugars as the major carbon source, such as glucose or sucrose, in this study, EPSs were induced from a squid pen powder (SPP)-containing medium by *Paenibacillus mucilaginosus* TKU032, a bacterial strain isolated from Taiwanese soil. Under the optimal culture conditions, the maximum EPS yield (14.8 g/L) was obtained. MALDI-TOF MS analysis of an EPS fraction purified by gel filtration revealed two mass peaks with molecular weights of ∼1.05 × 10^4^ and ∼1.35 × 10^4^ Da, respectively. The analysis of the hydrolysates of TKU032 EPS with cellulase, pectinase or α-amylase indicated that the glycosidic bond of TKU032 EPS is most likely an α-1,4 glycosidic bond and the hydrolysates are similar to those of starch. In addition, the purified EPS demonstrated strong antioxidant abilities.

## 1. Introduction

Free radicals are evidently harmful to living organisms [1]. To reduce the damage caused by free radicals, both synthetic and natural antioxidants are used. However, synthetic antioxidants are thought to cause liver damage and carcinogenesis [2]. Therefore, it is essential to develop natural nontoxic antioxidants to protect humans from free radicals. Novel natural antioxidants have gained great importance in science and medicine in recent decades.

Natural polysaccharides are highly susceptible to biodegradation and are less harmful than synthetic polymers [3,4]. Over the past few decades, the number of known exopolysaccharides (EPSs) produced by microbial fermentation has gradually increased. Microbial EPSs have many applications in the food and pharmaceutical industries, among others, as their physiological activities differ from those of natural gums and synthetic polymers [5,6]. However, antioxidant, anticancer, and immunoregulatory properties have been reported from many types of EPSs derived from filamentous fungi [7], such as *Cordyceps militaris* SU5-08 [7], *Fusarium solani* SD5 [1], *Pleurotus sajor-caju* [8], *Fomes fomentarius* [9], *Tremella fuciformis* [10], *Agrocybe cylindracea* [11], *Collybia maculate* [12], *Cordyceps jiangxiensis* [13] and *Tremella mesenterica* [14]. On the other hand, relative to fungal EPS, reports concerning the antioxidant activities of bacterial polysaccharides [15,16,17] are rarer.

In this study, a novel EPS-producing bacterium, *Paenibacillus mucilaginosus*, has been isolated from Taiwanese soil, and the EPS exhibited a significantly high level of antioxidant activity when squid pen was added into the liquid basal medium as the sole carbon/nitrogen source. Besides, EPS production from *P. mucilaginosus* has not been reported, and therefore, the aim of this study was to investigate optimal culture conditions to induce EPS production from this strain as well as to characterise the isolated EPS. We further investigated and evaluated the antioxidant activity of the purified EPS compared to a known antioxidant, such as ascorbic acid.

## 2. Results and Discussion

### 2.1. Isolation and Identification of the TKU032 Strain

To isolate microbial-derived EPSs, we investigated the amounts of total sugar from culture supernatants using squid pen as the sole carbon/nitrogen source. Among the more than 200 strains obtained, isolated in the laboratory and screened for EPS production, the TKU032 strain was selected. The TKU032 strain that showed maximal EPS content was isolated, maintained on nutrient agar and used throughout the study. According to the results of 16S rDNA nucleotide sequence analysis, strain TKU032 shows 99% similarity to *Paenibacillus mucilaginosus* (accession number NC_017672.1). TKU032-derived 16S rDNA sequence information most closely aligned with that of *Paenibacillus mucilaginosus*.

### 2.2. Culture Conditions for EPS Production

In our preliminary experiments, both SPP and SHP were used separately as the sole carbon/nitrogen sources to investigate EPS production from *P. mucilaginosus* TKU032. The total sugar contents of the media after culturing at 37 °C for 4 days were 12.3 g/L and 9.5 g/L for media containing 1% (*w*/*v*) SPP and 1% (*w*/*v*) SHP, respectively. These results indicated that SPP was a better substrate for EPS production by TKU032. The effects of various factors, such as carbon source concentration, medium aeration, culture temperature, and culture pH, were consecutively evaluated in single-factor experiments to establish optimal conditions for EPS production from *P. mucilaginosus* TKU032.

#### 2.2.1. Effect of SPP Concentration

**Figure 1:**
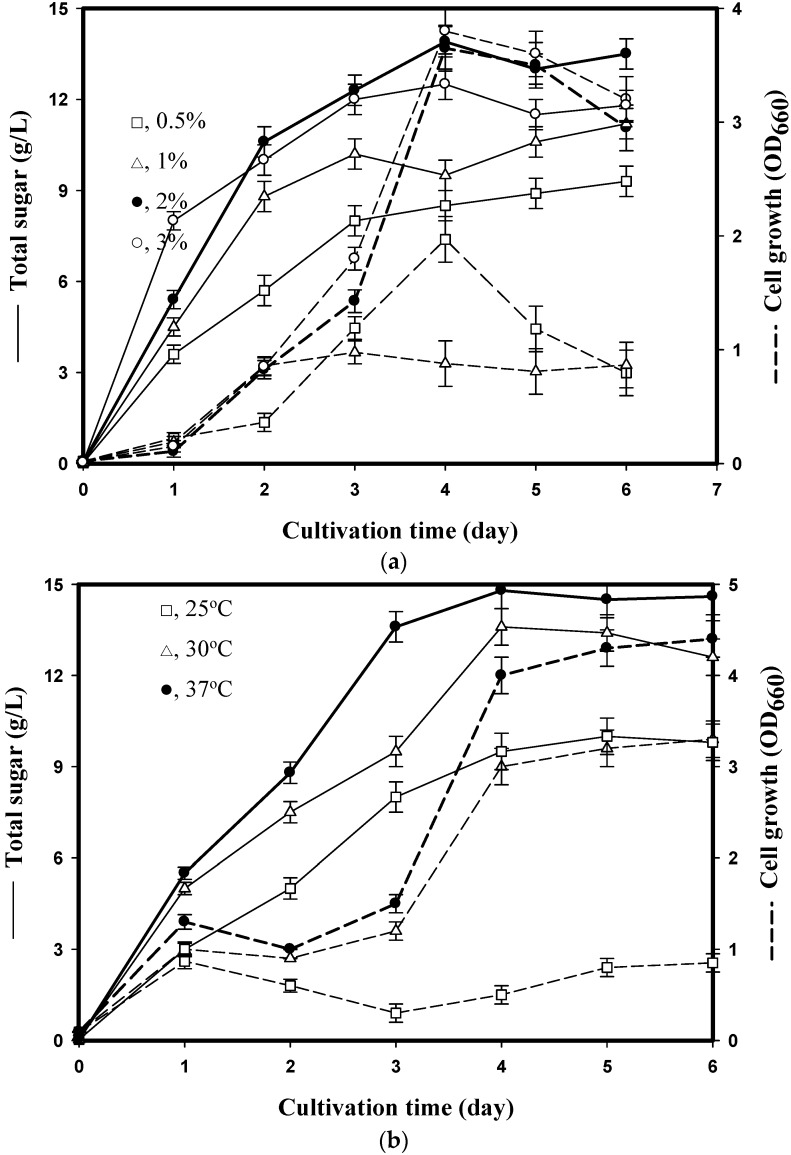
Effects of squid pen powder (SPP) suspension (**a**) and culture temperature (**b**) on cell growth (dashed line) and exopolysaccharides (EPS) (solid line) production by *P. mucilaginosus* TKU032. All data points are means ± S.D. (standard deviation) of three different experiments performed on different days (each experiment was conducted in triplicate).

Previous EPS studies have indicated that the medium composition plays a critical role in EPS production [2,18]. To select the optimal SPP concentration for EPS production, 0.5%–3% (*w*/*v*) SPP was added to the basal medium. Bacteria were cultured with medium containing 0.5%–3% (*w*/*v*) SPP at 37 °C for 6 days. The highest amount of total sugar (13.9 g/L) was obtained at 2% (*w*/*v*) SPP (Figure 1a). The bacteria grew rapidly during the first 4 days, and we also found that total sugar content was closely related to cell growth (Figure 1a). The total sugar yield reached its maximum level after cell growth had reached the stationary phase on the 4th day. These results indicate that 2% (*w*/*v*) SPP was most suitable for EPS production from *P. mucilaginosus* TKU032 and that the production of EPS is cell growth dependent. In addition, *P. mucilaginosus* TKU032 is a promising source of EPS.

#### 2.2.2. Effect of Culture Volume and Temperature

The effect of different culture medium volumes in 250-mL Erlenmyer flasks on EPS production by *P. mucilaginosus* TKU032 was investigated. TKU032 was inoculated in the medium described above containing 2% (*w*/*v*) SPP. The total sugar content was 14.8, 14.3, 13.8 and 14.1 g/L for culture volumes of 50, 100, 150 and 200 mL, respectively. Therefore, 50 mL of medium was more suitable for EPS production than 100, 150 or 200 mL.

Incubation temperature is another critical factor for EPS biosynthesis [2]. In a preliminary step, *P. mucilaginosus* TKU032 was cultured at three different temperatures, 4, 25 and 55 °C, to respectively represent psychrophilic, mesophilic and thermophilic conditions. It was found that *P. mucilaginosus* TKU032 belonged to the mesophilic bacterium. Moreover, *P. mucilaginosus* TKU032 was isolated from soils and the temperature of the environment is below 37 °C. Consequently, bacteria were cultured in conical flasks under the conditions described above (2% (*w*/*v*) SPP, 50 mL, 150 rpm) for 6 days at various temperatures (25 °C, 30 °C, 37 °C). The optimal temperature for EPS production was 37 °C for 4 days at which point the total sugar content reached 14.8 g/L (Figure 1b).

#### 2.2.3. Effect of Initial pH

**Figure 2:**
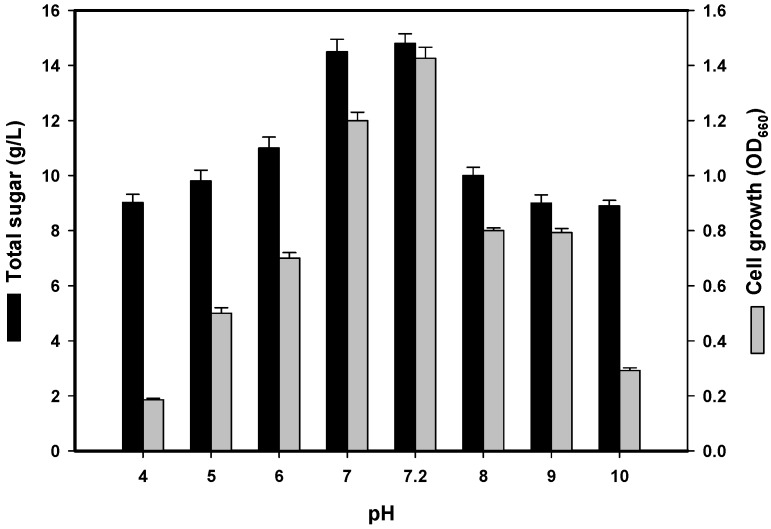
Effects of initial pH on cell growth and EPS production by *P. mucilaginosus* TKU032. All data points are means ± S.D. (standard deviation) of three different experiments performed on different days (each experiment was conducted in triplicate).

The initial culture pH is also an important factor that may affect the cell membrane, morphology and structure as well as the uptake of various nutrients and the rate of EPS biosynthesis [2,11]. As shown in Figure 2, the optimal pH value for EPS production was 7.2 before sterilisation (*i.e*., unadjusted pH), which corresponded to a total sugar content of 14.8 g/L after cultivation. This result was similar to that observed from soil-derived *P. polymyxa* strains [19,20], but unlike the slightly alkaline (pH 8) conditions required for EPS production by *P. polymyxa* EJS-3 [2]. As a neutral pH was more suitable for TKU032 EPS production, subsequent experiments were conducted with an unadjusted pH of 7.2.

#### 2.2.4. Time Course of EPS Production

The use of SPP as the sole C/N source for the EPS production was investigated. As shown in Figure 1b, the maximum total sugar content (14.8 g/L) was observed in a 50-mL culture incubated at 37 °C for 4 days. After this time point, the sugar content began to decrease. The bacteria grew rapidly during the first 4 days of culture, and we also found that the total sugar content was closely related to cell growth. The EPS yield (14.8 g/L) reached its maximum level after 4 days, at which point cell growth reached the maximum of the exponential phase (Figure 1b). This result indicates that EPS production is cell growth dependent and that *P. mucilaginosus* TKU032 is a promising EPS producer. No decrease in EPS production between day 4–6 and no further synthesis of EPS after day 4 (Figure 1b), probably because of substrate depletion and need for medium replacement. Among *Paenibacillus* spp., *P. polymyxa* EJS-3 produced EPSs when cultured on 16% sucrose and 1% yeast extract as the carbon and nitrogen source [2]. Compared to *P. polymyxa* EJS-3, EPS production by *P. mucilaginosus* TKU032 used a less expensive medium, SPP as the sole carbon/nitrogen source. The production of inexpensive EPS is an important factor in the utilisation of fishery waste products. The discovery of an inexpensive EPS not only solves environmental problems but also promotes the economic value of marine waste. Besides, *P. mucilaginosus* TKU032 adjusted to the culture conditions and could use fish waste SPP as a C/N source to produce EPSs. The EPS yield (14.8 g/L) of TKU032 was markedly higher than that of other microbes, such as *Pseudomonas oleovorans* NRRL B-14682 (11.82 g/L) [21], *Paecilomyces tenuipes* C240 (2.36 g/L) [22], *Rhodotorula acheniorum* MC (2 g/L) [23] and *Halomonas eurihalina* H212 (1.6 g/L) [24].

### 2.3. Isolation and Molecular Weight Determination of TKU032 EPS

**Figure 3:**
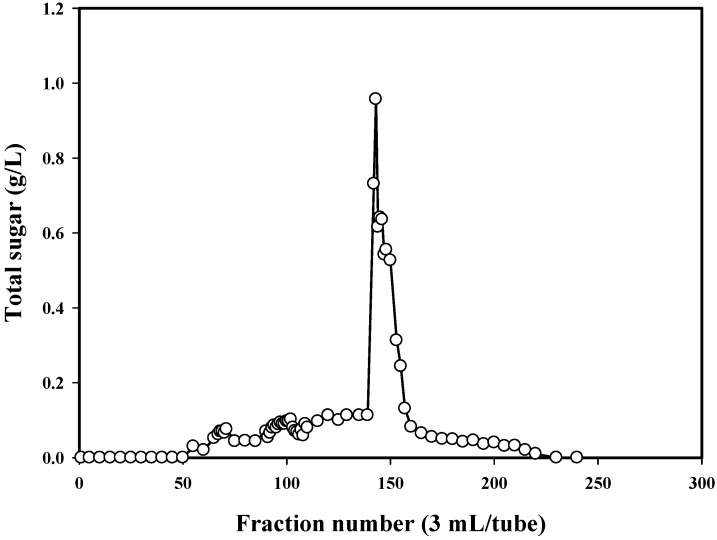
Elution profile of the EPS on Sephacryl S-100.

**Figure 4:**
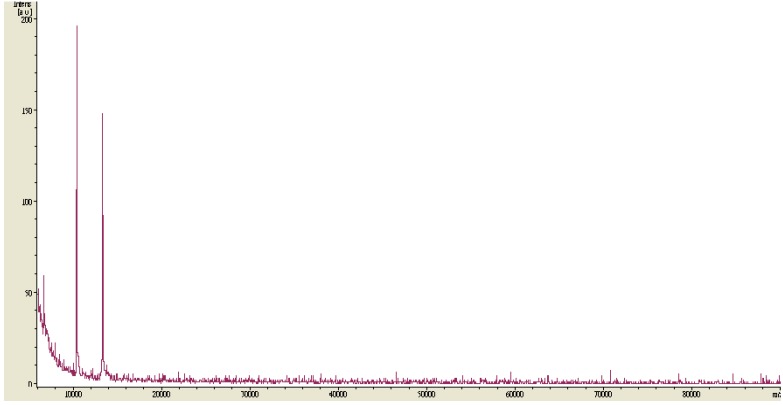
MALDI-TOF-MS of the EPS fraction obtained after gel filtration chromatography.

*P. mucilaginosus* TKU032 was grown in the optimal media as above mentioned. The fermented broth was centrifuged and EPS was precipitated in ethanol. The precipitated materials were dialysed before freeze drying and deproteinization with Sevag reagent (CHCl_3_-BuOH, *v*/*v* = 5/1) to obtain deproteinised polysaccharide. The water-soluble deproteinised polysaccharide was finally purified by gel filtration chromatography using a Sephacryl S-100 column (Figure 3). The EPS fractions of the one peak containing high concentration of total sugar were collected for further investigation. MALDI-TOF analysis of the EPS fraction was completed, and the resultant mass spectrum contained an x axis representing *m/z* (mass divided by charge) and a y axis representing absolute intensity (the number of ions of each species that reach the detector). The MALDI-TOF MS of the EPS fraction revealed two mass peaks with molecular weights of ∼1.05 × 10^4^ and ∼1.35 × 10^4^ Da, respectively (Figure 4).

### 2.4. Analysis of EPS Hydrolysates

**Figure 5:**
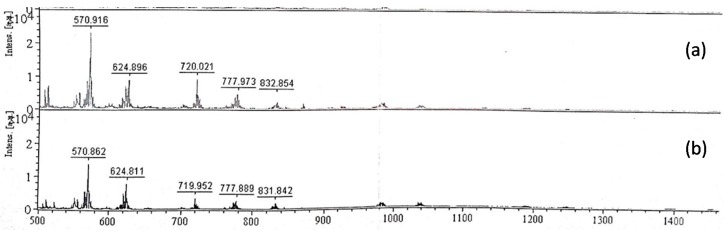
MALDI-TOF-MS of hydrolysates obtained from the hydrolysis of TKU032 EPS (**a**) and starch (**b**) with α-amylase.

Purified EPS samples were hydrolysed with 0.5 U/mL cellulase, pectinase or α-amylase at 45 °C for 24 h to estimate the glycosidic bond nature of each sample from the hydrolysates. The hydrolysates obtained were separated with TLC. No hydrolysis was observed for EPSs treated with pectinase and cellulase, whereas EPSs treated with α-amylase showed three bands on the TLC plates (data not shown). The difference in EPS hydrolysis by cellulase and α-amylase implied that the glycosidic bond is most likely an α-1,4 glycosidic bond rather than a β-1,4 glycosidic bond. Compared with the hydrolysates of starch by α-amylase, the MALDI-TOF MS of the TKU032 EPS hydrolysates was similar to that of starch (Figure 5).

### 2.5. Antioxidant Activity Study

Antioxidant activities have been attributed to various reactions and mechanisms. In this experiment, the *in vitro* antioxidant capacities of EPS were evaluated using the DPPH radical scavenging assay and reducing power analysis.

#### 2.5.1. DPPH Radical Scavenging Activity Assay

**Figure 6:**
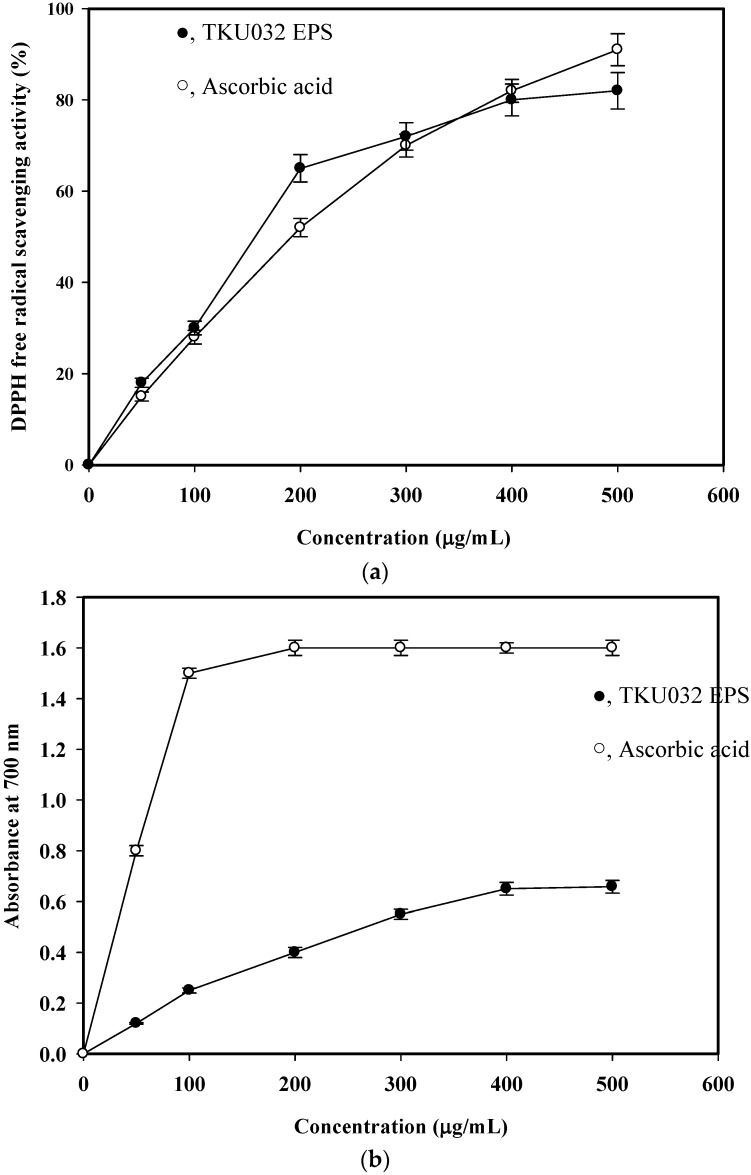
DPPH free radical scavenging ability (**a**) and reducing power (**b**) of EPS fraction from *P. mucilaginosus* TKU032. All data points are means ± S.D. (standard deviation) of three different experiments performed on different days (each experiment was conducted in triplicate).

The *in vitro* antioxidant activity of the isolated EPS was determined by the DPPH free radical scavenging ability. DPPH is one of the compounds that has a proton free radical with a characteristic absorption, which decreases significantly on exposure to proton radical scavengers [25]. Furthermore, it is well accepted that the DPPH free radical scavenging by antioxidants is due to their hydrogen-donating ability. The present findings showed that EPS isolated from *P. mucilaginosus* TKU032 had a noticeable DPPH free radical scavenging activity (Figure 6a). It was also observed that the DPPH scavenging activity was increased in a dose dependent (0–400 μg/mL) manner. It was assumed that the isolated EPS somehow donates hydrogen ions to react with the DPPH radical. The greatest scavenging rate of TKU032 EPS was 80%, which was higher than the 72% observed for *Serratia ureilytica* TKU013 [26] and 77% for *Paenibacillus* sp. TKU023 [25]. The half maximal effective concentration (EC_50_) of TKU032 EPS (157.1 μg/mL) was much lower than that of the standard antioxidant ascorbic acid (191.7 μg/mL) (Figure 6a). TKU032 EPS was a potent and natural antioxidant that could be used as an alternative to synthetic antioxidants.

#### 2.5.2. Reducing Power

During the reducing power assay, the capability of antioxidant compounds to reduce the Fe^3+^/ferricyanide complex to its ferric form is monitored by absorbance of Perl’s Prussian blue formation at 700 nm [26]. The reducing powers of TKU032 EPS are shown in Figure 6b. The reducing power (absorbance at 700 nm) of TKU032 EPS at a dosage of 400 μg/mL was 0.65 and did not increase with concentration. The reducing powers of TKU032 EPS were lower than that of ascorbic acid. TKU032 EPS was a good electron donor and can terminate the radical chain reactions by converting free radicals to more stable products.

The antioxidant activity of a polysaccharide depends chiefly on its structural characteristics, including molecular weight, monosaccharide content, and configuration of the glycosidic bond, among others, and is thus not a function of a single factor but a combination of several factors [27]. The MALDI-TOF MS of TKU032 EPS hydrolysates were similar to those of starch hydrolysates. Glucose may be a major monosaccharide component in TKU032 EPS. The greater glucose content in the polysaccharide accounted for its differential antioxidant properties [28]. Zhang *et al.*, found that a water-soluble polysaccharide with the molecular weight in the range of 40–80 × 10^4^ from *Pleurotus tuberregium* had a higher antitumor activity than those with molecular weights below 5 × 10^4^ [29]. However, TKU032 EPS has a relatively lower molecular weight, and deserves further investigation as to whether the lower EPS molecule containing high glucose content from bacteria also has relatively higher antioxidant activity.

## 3. Materials and Methods

### 3.1. Materials

Squid pen powder (SPP) and shrimp head powder (SHP) were prepared as previously described [30]. Squid pen and shrimp head powders were purchased from the Shin-Ma Frozen Food Co. (I-Lan, Taiwan), washed thoroughly with tap water and then dried. The dried materials were ground to a powder for use as the carbon/nitrogen source during EPS production. All other reagents used were of the highest grade available.

### 3.2. Screening and Identification of Microorganisms

The microorganisms were isolated from soil samples collected at different locations in northern Taiwan. One gram of soil was ground in a porcelain mortar, 10 mL of sterile distilled water was then added, and the soil suspension was stirred. The soil was eliminated by centrifugation gently, and the supernatant was centrifuged to harvest the suspended cells. The cell pellet harvested was spread on basal agar medium containing 1% SPP to obtain the various heterotrophic bacteria. The organisms obtained from this screening were subcultured in liquid media (containing 1% SPP, 0.1% K_2_HPO_4_ and 0.05% MgSO_4_·7H_2_O) in shaking flasks at 30 °C on a rotary shaker (150 rpm, Yih Der LM-570R). After incubation for 2 days, the culture broth was centrifuged (4 °C at 12,000 *g* for 20 min, Kubota 5922), and the supernatants were collected for the measurement of total sugar content using the procedure described below. The strain showed the highest total sugar content was selected and designated as TKU032.

Morphological, physiological and biochemical analysis as well as 16S rDNA sequencing after PCR amplification and cloning were used to identify the bacterial strain TKU032. The DNA sequences obtained were compiled and compared with those in the GenBank database using the BLAST program.

### 3.3. Culture Conditions for EPS Production

#### 3.3.1. Concentration of Carbon/Nitrogen Sources

SPP (0.5–2 g) was added to 100 mL of basal medium (containing 0.1% K_2_HPO_4_ and 0.05% MgSO_4_·7H_2_O). Cultures were inoculated with *P. mucilaginosus* TKU032 and incubated at 37 °C with agitation at 150 rpm for 6 days. At the conclusion of the growth period, the fermentation broth was centrifuged and total sugar content was assessed. After analysis, the carbon/nitrogen source concentrations that enabled maximal EPS production were identified. Optimal culture volume for maximal EPS production was then investigated.

#### 3.3.2. Culture Volume

The degree of aeration in the culture medium affects cell growth, and aeration is influenced by culture volume. Thus, a medium containing 2% (*w/v*) SPP, 0.1% K_2_HPO_4_, and 0.05% MgSO_4_·7H_2_O was used to investigate the relationship between medium volume and EPS production. Different volumes (50, 100, 150 and 200 mL) of medium were poured into individual 250-mL flasks. Bacterial liquid cultures were grown at 37 °C with agitation at 150 rpm for 6 days to study the effect of medium volume on TKU032 EPS production. Culture volumes that resulted in the greatest EPS production were selected.

#### 3.3.3. Culture Temperature and Medium pH

Conical flasks containing 50 mL of medium (consisting of 2% (*w*/*v*) SPP, 0.1% K_2_HPO_4_ and 0.05% MgSO_4_·7H_2_O) were used to investigate the influence of various temperatures (25 °C, 30 °C, or 37 °C) on TKU032 EPS production. The temperature most favourable for EPS production was selected. To determine the optimum initial medium pH for TKU032 EPS production, the medium was adjusted to the appropriate pH by the addition of 1 N HCl or 1 N NaOH prior to sterilisation. A time-course experiment was performed in a 250-mL flask containing the optimised culture medium based on the results of the single-factor experiments.

### 3.4. Total Sugar Measurement

The phenol-sulphuric acid method was used to evaluate the total sugar present in the medium [31]. Briefly, 25 μL of 5% phenol was added to 1 mL of growth medium. After shaking, 2.5 mL of concentrated H_2_SO_4_ was added. The mixture was incubated at room temperature for 10 min, and the absorbance was read at 490 nm with pure d-glucose used as a standard.

### 3.5. Isolation of TKU032 EPS

After fermentation, the sample was immediately autoclaved for 20 min to reduce accumulated bacterial debris and centrifuged (12,000 *g* for 20 min) to remove the remaining SPP and biomass. The supernatant was mixed with two volumes of ethanol, stirred vigorously and stored overnight at 4 °C. The precipitate from the ethanol dispersion was collected by centrifugation at 12,000 *g* for 15 min, re-dissolved in distilled water and lyophilised to yield the crude EPS.

### 3.6. Deproteinisation of TKU032 EPS

The crude EPS was re-dissolved in distilled water, vigorously stirred at 80 °C for 30 min, mixed with four volumes of anhydrous ethanol, vigorously stirred again and stored overnight at 4 °C. The precipitate from the ethanol dispersion was collected by centrifugation at 12,000 *g* for 15 min, re-dissolved in distilled water and deproteinised with 1/5 volume of Sevag reagent (CHCl_3_-BuOH, *v*/*v* = 5/1) seven times [32]. The deproteinised solution was then dialysed against distilled water, concentrated and lyophilised to yield deproteinised EPS.

### 3.7. Purification of TKU032 EPS

The deproteinised EPS was purified sequentially by Sephacryl S-100 chromatography. The deproteinised EPS solution (10 mg/mL, 3 mL) was applied to a Sephacryl S-100 column (2.5 × 100 cm). Distilled water eluate (5 mL/tube) was collected automatically, and the carbohydrate content was determined using the phenol-sulphuric acid method with glucose as a standard [31]. The polysaccharide fractions obtained were then pooled, concentrated and lyophilised for further study.

### 3.8. MALDI-TOF MS Analysis

One microliter of the sample solution (2 mg/mL) was mixed on the target with 1 μL of 2,5-dihydroxybenzoic acid as a matrix (15 mg/mL) in H_2_O-ACN-TFA (50/50/0.1%, *v*/*v*/*v*). Positive ion MALDI mass spectra were acquired with a MALDI-TOF instrument (Bruker Daltonics, Bremen, Germany) equipped with a nitrogen laser emitting at 337 nm and operating in linear mode. Each mass spectrum represented approximately 30–50 laser shots. External 3-point calibration was used for mass assignment.

### 3.9. Analysis of EPS hydrolysates

For monosaccharide composition analysis, the deproteinised EPS (25 mg) samples were dissolved in 50 mL of phosphate buffer (pH 6) and hydrolysed with 0.5 U/mL of cellulase, pectinase or α-amylase at 45 °C for 24 h. The hydrolysates were then dialysed against distilled water, concentrated and lyophilised. The hydrolysates were analysed by silica gel thin layer chromatography (TLC) using a developing solvent of *n*-butanol:ethanol:water (2:1:1, *v*/*v*/*v*). Silica gel TLC plates (0.25 mm) were obtained from E. Merck. After TLC plate development, carbohydrates were visualised by spraying TLC plates with 5% (*v*/*v*) sulphuric acid in ethanol and heating the plates. Glucose, galactose and mannose were used as standard monosaccharides. The hydrolysates of TKU032 EPS were further analysed by MALDI-TOF MS.

### 3.10. Antioxidant Activity Assays

#### 3.10.1. Measurement of DPPH Radical Scavenging Activity

The diluted EPS solution (150 αL) was mixed with 37.5 αL of a methanolic solution containing 0.75 mM DPPH. The mixture was shaken vigorously and incubated at room temperature for 30 min in the dark, and the absorbance at 517 nm was then measured and normalised to a blank sample [21]. The scavenging ability was calculated using the following equation: Scavenging activity (%) = [(*A*_517_ of control − *A*_517_ of sample)/*A*_517_ of control] × 100.

#### 3.10.2. Measurement of Reducing Power

A method developed by Oyaizu [33] for testing reducing power was used. The diluted EPS solution or distilled water (control) (0.5 mL) was mixed with 0.5 mL of sodium phosphate buffer (0.02 M, pH 7) and 0.5 mL of 1% potassium ferricyanide. The mixture was then incubated in a 50 °C water bath for 20 min. The resulting solution was rapidly cooled, mixed with 0.5 mL of 10% trichloroacetic acid, and centrifuged at 800 *g* for 10 min. The supernatant (1.5 mL) was then mixed with 0.2 mL of 0.1% ferrichloride. After allowing the reaction to proceed for 10 min, the absorbance at 700 nm was measured [26]. Higher absorbance values indicated greater reducing power.

## 4. Conclusions

In the present study, the optimum culture conditions for novel EPS production by *P. mucilaginosus* TKU032 were investigated. The EPS was produced by *P. mucilaginosus* TKU032 using SPP as the sole carbon/nitrogen source and was purified by gel filtration chromatography. One EPS fraction was characterised by MALDI-TOF analysis, and its antioxidant activity subsequently investigated. The *in vitro* antioxidant assay showed strong antioxidant properties, especially DPPH free radical scavenging ability. These results suggest that EPS from *P. mucilaginosus* TKU032 may be a natural alternative to conventionally used products with potential antioxidant activity. These findings appear to be useful for further research aiming to analyze the composition of TKU032 EPS by unspecific degradation with acids and identification of liberated monosaccharides.
